# Supplementary material for: Mycobacterium abscessus Genetic Determinants Associated with the Intrinsic Resistance to Antibiotics
Source: Microorganisms. 2021 Dec 7;9(12):2527. doi: 10.3390/microorganisms9122527 (PMC8707978; doi:10.3390/microorganisms9122527)
Supplement: Supplementary file 1 [file microorganisms-09-02527-s001.zip › microorganisms-1460163-supplementary.pdf]

Supplemental Table S1. *M. abscessus* 19977 gene knockout mutants associated with increased (BC) or decreased (MIC) susceptibility to antibiotics and categorized into functional groups.

Gene	Function	Conserved Domain/Notes	MIC/BC
**Cell Wall and Transport Mechanisms**			
**MAB_0734**	MspA membrane porin	Contains a signal peptide.	BC
**MAB_0937c**	MmpL membrane drug exporter protein	Transport protein with a role in drug resistance.	MIC
**MAB_1137c**	Putative MmpL membrane protein	Possible role in drug resistance.	MIC
**MAB_1170**	Probable membrane transporter protein.	TauE, Sulfite exporter. Integral membrane protein involved in the transport of anions across the cytoplasmic membrane during taurine metabolism as an exporter of sulfoacetate.	MIC
**MAB_1171c**	Conserved hypothetical protein	Integral membrane transporter protein	MIC
**MAB_1315**	Putative lipoprotein LpqW	Synthesis and transport of the unique components of the mycobacterial cell wall	MIC
**MAB_1352**	Probable glucose-1-phosphate adenylyltransferase	glgC glucose-1-phosphate adenylyltransferase	BC
**MAB_1418**	Uncharacterized protein	Lprb lipoproteins biofilm formation	MIC
**MAB_1632c**	Conserved hypothetical protein	DUF2339 superfamily: Predicted membrane protein	MIC
**MAB_1839**	Diguanylate cyclase/phosphodiesterase	GGDEF Diguanylate-cyclase/ABC2_membrane superfamily regulates cell surface adhesion in bacteria/ABC-2 type transporter	MIC
**MAB_2421c**	Hypothetical protein	PknH_C like extracellular domain	BC
**MAB_2435**	Molybdenum ABC transporter ModC, ATP-binding protein	P-loop_NTPase superfamily	MIC
**MAB_2553**	Putative integral membrane protein		BC

**MAB_2776**	Membrane protein/LPBR	Potential membrane protein with signal peptide	MIC
**MAB_2785**	Hypothetical protein	Rhamnose ABC transporter, rhamnose transport	MIC
**MAB_2787c**	Probable molybdenum ABC transporter, periplasmic	Periplasmic binding protein type 2 superfamily	MIC
**MAB_2919c**	Probable dipeptidyl peptidase IV	Amino acid transport and metabolism	MIC
**MAB_3384c**	Putative ABC transporter, ATP-binding protein	ModF ABC-type molybdenum transport system	MIC
**MAB_3465**	Putative sulfate transporter/antisigma-factor		MIC
**MAB_3682c**	Conserved hypothetical protein	Virul_fac_BrkB superfamily: conserved inner membrane protein	MIC
**MAB_4036**	Conserved hypothetical protein	ABC_6TM_exporters superfamily: Six-transmembrane helical domain of an uncharacterized ABC exporter	MIC
**MAB_4117c**	Putative membrane protein	MmpS family protein, has a potential role in the transport of MmpL substrates.	BC
**MAB_4237c**	Putative amino acid ABC transporter, ATP-binding protein	GlnQ	MIC
**MAB_4691c**	Probable non-ribosomal peptide synthetase PstA	Sulfate Transporter and Anti-Sigma factor antagonist.	MIC
**MAB_4706c**	Conserved hypothetical protein	YeiB: Uncharacterized membrane protein	MIC
**MAB_4915c**	Hypothetical protein	PknH_C like extracellular domain	BC

**Regulatory Proteins**			
**MAB_0068**	Putative transcriptional regulator, GntR family	Regulate transporters, antibiotic biosynthesis, and cell permeability	MIC

**MAB_0161**	Probable transcriptional regulator, LysR family	Antimicrobial resistance, efflux pump regulator, oxidative stress response regulator	MIC
**MAB_0756c**	Conserved hypothetical protein	AcrR: DNA-binding transcriptional regulator	MIC
**MAB_1589**	Putative transcriptional regulator, TetR family		MIC
**MAB_1881c**	Putative transcriptional regulator, TetR family	DNA-binding transcriptional regulator	MIC
**MAB_1926**	Putative transcriptional regulatory protein PrrA	OmpR DNA-binding response regulator	BC
**MAB_2057**	Conserved hypothetical protein	Hot_dog superfamily: fatty acid metabolism, transcriptional regulation of fatty acid biosynthesis	MIC
**MAB_2061c**	Putative transcriptional regulator, TetR family		MIC
**MAB_2458c**	Putative transcriptional regulator, TetR family. AcrR	TetR family. AcrR, DNA-binding transcriptional regulator, AcrR family [Transcription]	BC
**MAB_3318**	Putative regulatory protein.	ARSR subfamily, Arsenical Resistance Operon Repressor, and similar prokaryotic, metal regulated homodimeric repressors.	BC
**MAB_3345c**	NAD+-dependent DNA Ligase		BC
**MAB_4118**	Probable transcriptional regulatory protein	COG3800	MIC
**MAB_4710c**	Putative transcriptional regulator, TetR family	DUF1956 superfamily	MIC

**Information Pathways**			
**MAB_0279c**	Probable DNA ligase LigC	DNA replication and recombination	MIC
**MAB_0628**	Conserved hypothetical protein/MinD-like ATPase	involved in cell cycle control, cell division, chromosome partitioning, Cell motility	MIC

**MAB_0881**	Hypothetical tRNA/rRNA methyltransferase		MIC
**MAB_0977c**	Putative DNA-directed DNA polymerase		MIC
**MAB_1248**	Probable RNA polymerase sigma factor		MIC
**MAB_1583**	ATP-dependent Clp protease ATP-binding subunit ClpX		MIC
**MAB_2412c**	Probable IMP dehydrogenase family protein	important for DNA and RNA synthesis, signal transduction, energy transfer, glycoprotein synthesis	MIC
**MAB_3085c**	Thymidylate synthase ThyX	catalyzes the conversion of deoxyuridine monophosphate (dUMP) to deoxythymidine monophosphate (dTMP).	MIC
**MAB_3475c**	Putative cell division ATP-binding protein FtsE		MIC
**MAB_3543c**	RNA polymerase sigma-E factor		MIC
**MAB_3651**	Probable ATP-dependent helicase Lhr		MIC
**MAB_4341**	Conserved hypothetical protein	LigD Eukaryotic-type DNA primase: DNA replication, recombination, and repair	MIC
**MAB_4401**	Putative surface layer protein	DNA-binding beta-propeller fold protein YncE	MIC
**MAB_4738c**	Putative amidohydrolase	hydrolase that acts upon amide bonds	MIC
**MAB_4847c**	Putative helicase		MIC
**tRNA-Ala(CGC)**	Resolvase, partial	TB: Rv2187 fadD15 Probable long-chain-fatty-acid-CoA ligase (42%)	MIC

**Oxidation-Reduction Enzymes**			
**MAB_0463c**	Probable oxidase (copper-binding protein)	Oxidation Reduction Reaction	MIC

**MAB_0890c**	Putative homogentisate 1,2-dioxygenase	Breakdown of the amino acid's tyrosine and phenylalanine	MIC
**MAB_1866c**	Putative oxygenase	Smoa_sbd superfamily: Styrene monooxygenase	BC
**MAB_2053**	Luciferase-like, Flavin_utilizing_monoxygenases superfamily	Incorporate one hydroxyl group into the substrate, the oxidation reaction	MIC
**MAB_2075**	Putative monooxygenase	CzcO Predicted flavoprotein	BC
**MAB_2075**	Putative monooxygenase	CzcO Predicted flavoprotein	BC
**MAB_2449**	Flavin_utilizing_monoxygenases superfamily	Incorporate one hydroxyl group into a substrate, an oxidation reaction	MIC
**MAB_3693c**	Possible oxidoreductase	catalyzes the reduction of flavin or nitro compounds, Oxidation-Reduction Reaction	MIC
**MAB_3870c**	Probable molybdopterin oxidoreductase. Anaerobic selenocysteine-containing dehydrogenase	Oxidation-Reduction Reaction	MIC
**MAB_3870c**	Probable molybdopterin oxidoreductase	Molybdopterin-Binding (MopB) domain	BC
**MAB_3939 /MAB_3940c**	Conserved hypothetical protein/GGDEF domain-containing protein, deazaflavin-dependent oxidoreductase, nitroreductase family play roles in eukaryotic signal transduction and prokaryotic sensory pathways.	Oxidation-Reduction Reaction	MIC
**MAB_4055c**	Alpha/beta hydrolase fold	AB hydrolase 1: possible oxygenase, class of oxidoreductase	MIC

**Intermediary Metabolism and Respiration**

**MAB_0121**	Rhodanese-like protein	RHOD, stress response proteins phosphatase, protection from oxidants	MIC
**MAB_0164**	Probable short-chain dehydrogenase/reductase	cholesterol oxidation, sterol oxidation, universal stress protein, conserved across mycobacterium	MIC
**MAB_0177**	Antigen 85-A/B/C precursor	S-formylglutathione hydrolase, defense mechanisms, involved in lipid metabolism	MIC
**MAB_0178**	Abhydrolase superfamily Cutinase	degrade cutin a protective layer for lipids, involved in lipid metabolism of bacteria	MIC
**MAB_0179**	Probable fatty-acid-CoA ligase FadD	PRK07769:	BC
**MAB_0304**	oxoglutarate decarboxylase/oxoglutarate dehydrogenase	Cleave carbon-carbon bonds, Involved in Citric Acid Cycle	MIC
**MAB_0593c**	Probable acyl-CoA dehydrogenase FadE		MIC
**MAB_0820c**	Probable NAD-glutamate dehydrogenase	GDH nitrogen assimilation, catabolism of glutamate	MIC
**MAB_0823**	Probable aldehyde dehydrogenase	The enzyme involved intermediary metabolism and respiration pathways, help with dormancy, general stress resistance	MIC
**MAB_0959**	Probable enoyl-CoA hydratase/isomerase	PRK07854 enoyl-CoA hydratase	BC
**MAB_1194**	Putative lipase LipU		MIC
**MAB_1393c**	Probable 2-oxoglutarate dehydrogenase SucA	Involved in the Citric Acid cycle	MIC
**MAB_1517c**	Probable O-methyltransferase Omt	involved in polyketide biosynthesis	MIC
**MAB_1530**	Probable conserved lipoprotein LppS		MIC

**MAB_1637**	Transglutaminase-like	YebA Transglutaminase, enzymes that in nature primarily catalyze the formation of an isopeptide bond between γ-carboxamide groups of glutamine residue side chains and the ε-amino groups of lysine residue side chains with subsequent release of ammonia	BC
**MAB_1865**	Probable fatty-acid-CoA ligase FadD	lipid transport and metabolism	MIC
**MAB_1869c**	Probable zinc-dependent alcohol dehydrogenase AdhE2		MIC
**MAB_2042c**	Conserved hypothetical protein	PRK08257 acetyl-CoA acetyltransferase	BC
**MAB_2080**	Probable medium-chain fatty-acid-CoA ligase FadD.	PRK06187; long-chain-fatty-acid CoA ligase. This enzyme mediates Intrinsic Pyrazinamide Resistance in *M. tuberculosis*.	BC
**MAB_2112**	Hypothetical protein	Med15 superfamily Mediator complex non-fungal: regulation of cholesterol and fatty acid homeostasis.	MIC
**MAB_2257**	Probable polyketide synthase		MIC
**MAB_2797c**	Putative riboflavin synthase alpha chain	Lumazine-binding catalyzes the final step in riboflavin biosynthesis	MIC
**MAB_2979**	Conserved hypothetical protein (peptide methionine sulfoxide reductase-related protein)	Methionine sulfoxide reduction, cope with oxidative stress	MIC
**MAB_3010**	Hypothetical protein	MgATP-dependent phosphorylation of a variety of sugar substrates.	MIC
**MAB_3040c**	Probable acyl-CoA dehydrogenase		MIC
**MAB_3281**	Putative enoyl-CoA hydratase/isomerase		BC

**MAB_3344**	Probable glycosyl transferase.ArnT;	transfer of saccharide moieties from an activated nucleotide sugar to a nucleophilic glycosyl acceptor molecule, 4-amino-4-deoxy-L-arabinose transferase, or related glycosyltransferase of PMT family [Cell wall/membrane/envelope biogenesis]	BC
**MAB_3659c**	Putative peptidase/amidohydrolase Cellular metabolism		BC
**MAB_3831c**	Putative glycosyltransferase	mycofact_glyco: mycofactocin system glycosyltransferase	BC
**MAB_4010c**	Conserved hypothetical protein	4HBT_3: a wide variety of enzymes, principally thioesterases, fatty acid synthesis	MIC
**MAB_4136c**	Aldehyde dehydrogenase	oxidize a wide range of endogenous and exogenous aliphatic and aromatic aldehydes to their corresponding carboxylic acids and play an important role in detoxification and stress response	MIC
**MAB_4171c**	NADPH-ferredoxin reductase FprA.	GltD; NADPH-dependent glutamate synthase beta chain or related oxidoreductase [Amino acid transport and metabolism, General function prediction only]	BC
**MAB_4455c**	Probable acyl-CoA synthetase FadD		MIC
**MAB_4868c**	Hypothetical protein	Acetyl-CoA carboxylase/COG3975 superfamily :Acetyl-CoA carboxylase, Predicted metalloprotease, fumarylacetoacetase	MIC

**Conserved Hypotheticals**			
**MAB_0177 /MAB_0176**	Antigen 85-A/B/C precursor/Antigen 85-A precursor	FrmB:	BC
**MAB_0516c**	PRK13798 putative OHCU decarboxylase		MIC
**MAB_0733**	Conserved hypothetical protein		MIC

**MAB_1022**	Conserved hypothetical protein (esterase?)		MIC
**MAB_1027**	Conserved hypothetical protein	Pfam15599: Immunity protein 63	MIC
**MAB_1116**	hypothetical protein/ metal-dependent hydrolase		MIC
**MAB_1418**	Hypothetical protein		BC
**MAB_1599**	Conserved hypothetical protein	Possible membrane protein	BC
**MAB_1822**	Conserved hypothetical protein	AbiJ_NTD4 super family	MIC
**MAB_2060**	Conserved hypothetical protein	acyclic terpene utilization (Atu) pathway	MIC
**MAB_2069**	Conserved hypothetical protein	metal-dependent hydrolase	MIC
**MAB_2104c**	Probable dihydroorotate dehydrogenase		MIC
**MAB_2197**	Probable precorrin-6X reductase CobK/Probable precorrin-4 C11-methyltransferase CobM		MIC
**MAB_2359**	Cytotoxin/hemolysin homolog TlyA	YqxC: Predicted rRNA methylase	BC
**MAB_2376c**	Conserved hypothetical protein		MIC
**MAB_2491c**	Conserved hypothetical protein	Tellurite resistance protein and related permeases	MIC
**MAB_2491c**	Conserved hypothetical protein	YddW superfamily	BC
**MAB_2618**	Uncharacterized Protein	DUF35_N	MIC
**MAB_3037c**	Conserved hypothetical protein	LysM	MIC
**MAB_3169c**	4-hydroxy-3-methylbut-2-en-1-yl diphosphate synthase(IspG)		MIC
**MAB_3185**	Hypothetical protein		MIC

**MAB_3822**	Hypothetical protein		MIC
**MAB_3822**	Hypothetical protein	AvrE super family cl26148, a Pathogenicity factor	BC
**MAB_3890c**	Probable histidine kinase response regulator	Fh1A GAF domain [Signal transduction mechanisms]	BC
**MAB_4033**	Putative Mce family protein.	Mtu_fam_mce, virulence factor Mce family protein.	BC
**MAB_4181**	Sulfates adenylate transferase, subunit 2 (CysD)	PRK05253	BC
**MAB_4549c**	hypothetical protein	HEXXH_Rv0185, putative metallohydrolase, TIGR04338 family.	BC
**MAB_4661**	Conserved hypothetical protein	VTT superfamily	MIC
**MAB_4727c**	hypothetical protein	Mrr_cat; Restriction endonuclease	BC
**MAB_4804**	Hypothetical protein		BC
**Bacteriophage Proteins**			
**MAB_0233**	Putative phage tail tape measures protein TMP	tolA cell envelope integrity inner membrane protein	MIC
**MAB_1785**	Bacteriophage protein/	Terminase_1 superfamily: bacteriophage proteins, terminase large subunit proteins.	MIC

Supplemental Table S2. The Real-Time qPCR for 16 genes of transport systems across 9 MAB clinical isolates.

Genes	Antibiotic	MAB 19977	NR 49093 strain DJO 44274	NR 44273 strain 4529	NR 442746 strain 4530	DNA00703	DNA01163	DNA01627	DNA01715	DNA01639
MAB_4691c	AMK	0.22 ± 0.25	1.17 ± 0.00	0.43 ± 0.25	0.65 ± 0.67	0.08 ± 0.04	0.16 ± 0.07	0.41 ± 0.12	1.64 ± 1.97	1.32 ± 0.37
	CLA	1.06 ± 1.36	1.09 ± 0.00	1.69 ± 0.51	0.84 ± 0.94	0.12 ± 0.06	0.96 ± 0.47	0.34 ± 0.36	0.29 ± 0.25	0.29 ± 0.08
MAB_4237c	AMK	0.48 ± 0.56	1.23 ± 0.82	0.54 ± 0.38	13.16 ± 17.70	4.68 ± 2.23	2.75 ± 1.13	172.75 ± 51.35	2.69 ± 0.82	2.83 ± 0.00
	CLA	0.25 ± 0.01	0.40 ± 0.03	1.10 ± 0.00	1.43 ± 1.50	2.05 ± 0.98	6.71 ± 3.31	21.71 ± 20.28	2.11 ± 0.00	5.93 ± 5.34
MAB_2435	AMK	0.14 ± 0.10	1.27 ± 0.15	0.76 ± 0.06	1.48 ± 1.09	0.20 ± 0.09	0.45 ± 0.18	0.70 ± 0.21	0.19 ± 0.19	0.48 ± 0.00
	CLA	0.04 ± 0.00	0.84 ± 0.23	0.83 ± 0.83	0.50 ± 0.71	0.09 ± 0.04	0.00 ± 0.00	0.38 ± 0.33	0.15 ± 0.00	565.08 ± 0.00
MAB_1839	AMK	2.91 ± 0.00	1.41 ± 0.22	0.61 ± 0.00	49.82 ± 59.10	3.59 ± 1.71	9.87 ± 4.05	3.19 ± 0.95	12.32 ± 6.63	1.48 ± 0.00
	CLA	0.70 ± 0.00	1.30 ± 0.00	0.24 ± 0.00	28.71 ± 39.15	1.99 ± 0.95	30.75 ± 15.17	3.12 ± 3.47	5.38 ± 5.94	23.59 ± 0.00
MAB_4036	AMK	0.28 ± 0.36	0.61 ± 0.22	0.22 ± 0.12	0.09 ± 0.01	0.00 ± 0.00	0.06 ± 0.02	0.04 ± 0.01	0.04 ± 0.01	1.53 ± 0.43
	CLA	4.90 ± 0.00	0.52 ± 0.00	0.67 ± 0.00	0.00 ± 0.00	0.01 ± 0.00	1.00 ± 0.00	0.12 ± 0.15	0.02 ± 0.01	0.62 ± 0.54
MAB_0734	AMK	17.07 ± 21.77	1.02 ± 0.00	0.50 ± 0.45	157.59 ± 26.79	27.17 ± 12.93	41.40 ± 16.98	72.91 ± 21.67	3.89 ± 3.37	0.42 ± 0.00
	CLA	19.76 ± 16.43	2.58 ± 0.00	2.91 ± 2.36	279.68 ± 193.33	33.19 ± 15.79	482.17 ± 237.95	47.47 ± 45.62	23.89 ± 23.00	370.44 ± 0.00
MAB_1171c	AMK	1.52 ± 0.56	1.36 ± 0.00	0.32 ± 0.11	4.50 ± 5.76	0.11 ± 0.05	0.23 ± 0.09	0.12 ± 0.04	1.19 ± 0.00	1.61 ± 1.35
	CLA	3.02 ± 0.00	1.14 ± 0.00	0.98 ± 0.42	1.00 ± 0.00	0.13 ± 0.06	0.31 ± 0.00	0.53 ± 0.72	0.45 ± 0.00	0.60 ± 0.00
MAB_1137c	AMK	13.65 ± 5.13	2.87 ± 2.40	2.86 ± 0.00	28.14 ± 10.27	3.00 ± 1.43	9.11 ± 3.74	7.58 ± 2.25	14.47 ± 13.00	0.57 ± 0.00

	**CLA**	6.54 ± 6.79	28.94 ± 36.87	0.05 ± 0.00	9.11 ± 7.59	0.77 ± 0.37	13.83 ± 6.83	2.15 ± 0.81	3.20 ± 2.60	33.55 ± 0.00
**MAB_2787c**	**AMK**	0.25 ± 0.16	3.27 ± 1.19	1.21 ± 0.00	0.25 ± 0.00	0.12 ± 0.06	1.52 ± 0.62	0.61 ± 0.18	0.06 ± 0.04	1.72 ± 1.36
	**CLA**	0.17 ± 0.00	1.46 ± 0.00	1.18 ± 0.00	0.22 ± 0.22	0.11 ± 0.05	0.24 ± 0.33	0.75 ± 0.88	0.35 ± 0.34	0.00 ± 0.00
**MAB_2421c**	**AMK**	0.40 ± 0.11	1.18 ± 0.25	0.45 ± 0.26	2.19 ± 1.94	0.21 ± 0.10	0.45 ± 0.18	1.54 ± 0.46	0.07 ± 0.05	0.22 ± 0.11
	**CLA**	0.39 ± 0.00	1.31 ± 0.15	0.65 ± 0.00	1.58 ± 1.67	0.12 ± 0.06	3.02 ± 1.49	0.87 ± 0.77	2.80 ± 2.86	1.11 ± 0.71
**MAB_3384c**	**AMK**	1.63 ± 0.00	0.69 ± 0.00	0.25 ± 0.16	2.56 ± 2.60	0.34 ± 0.16	0.64 ± 0.26	0.58 ± 0.17	0.27 ± 0.21	1.52 ± 0.83
	**CLA**	2.15 ± 0.00	0.21 ± 0.06	0.57 ± 0.00	2.01 ± 1.94	0.09 ± 0.04	2.80 ± 1.38	0.34 ± 0.31	0.20 ± 0.21	4.13 ± 5.42
**MAB_4117c**	**AMK**	0.20 ± 0.11	1.52 ± 0.09	1.09 ± 0.99	0.29 ± 0.34	0.08 ± 0.04	0.05 ± 0.02	0.09 ± 0.03	0.03 ± 0.00	1.24 ± 0.15
	**CLA**	6.29 ± 6.81	0.64 ± 0.01	2.26 ± 2.43	11.84 ± 4.09	2.58 ± 0.00	112.72 ± 92.50	2.50 ± 3.50	5.89 ± 0.00	3.16 ± 2.55
**MAB_3465**	**AMK**	14.57 ± 3.69	0.66 ± 0.15	0.31 ± 0.18	16.23 ± 12.00	2.26 ± 1.07	6.76 ± 2.77	5.85 ± 1.74	0.63 ± 0.36	0.92 ± 0.43
	**CLA**	22.78 ± 25.20	0.60 ± 0.40	0.63 ± 0.01	18.93 ± 13.00	1.26 ± 0.60	21.66 ± 10.69	6.08 ± 6.87	20.93 ± 20.50	38.37 ± 28.22
**MAB_4915c**	**AMK**	1.20 ± 0.41	0.76 ± 0.21	0.46 ± 0.40	0.28 ± 0.16	0.30 ± 0.14	0.28 ± 0.11	0.65 ± 0.19	0.30 ± 0.35	1.59 ± 0.62
	**CLA**	1.11 ± 0.65	1.52 ± 0.00	1.66 ± 0.02	1.07 ± 1.16	0.14 ± 0.07	6.15 ± 3.03	0.44 ± 0.43	0.09 ± 0.04	2.61 ± 2.56

Genes	Antibiotic	MAB 19977	NR 49093 strain DJO 44274	NR 44273 strain 4529	NR 442746 strain 4530	DNA00703	DNA01163	DNA01627	DNA01715	DNA01639
MAB_4691c	AMK	0.22 ± 0.25	1.17 ± 0.00	0.43 ± 0.25	0.65 ± 0.67	0.08 ± 0.04	0.16 ± 0.07	0.41 ± 0.12	1.64 ± 1.97	1.32 ± 0.37
	CLA	1.06 ± 1.36	1.09 ± 0.00	1.69 ± 0.51	0.84 ± 0.94	0.12 ± 0.06	0.96 ± 0.47	0.34 ± 0.36	0.29 ± 0.25	0.29 ± 0.08
MAB_4237c	AMK	0.48 ± 0.56	1.23 ± 0.82	0.54 ± 0.38	13.16 ± 17.70	4.68 ± 2.23	2.75 ± 1.13	172.75 ± 51.35	2.69 ± 0.82	2.83 ± 0.00
	CLA	0.25 ± 0.01	0.40 ± 0.03	1.10 ± 0.00	1.43 ± 1.50	2.05 ± 0.98	6.71 ± 3.31	21.71 ± 20.28	2.11 ± 0.00	5.93 ± 5.34
MAB_2435	AMK	0.14 ± 0.10	1.27 ± 0.15	0.76 ± 0.06	1.48 ± 1.09	0.20 ± 0.09	0.45 ± 0.18	0.70 ± 0.21	0.19 ± 0.19	0.48 ± 0.00
	CLA	0.04 ± 0.00	0.84 ± 0.23	0.83 ± 0.83	0.50 ± 0.71	0.09 ± 0.04	0.00 ± 0.00	0.38 ± 0.33	0.15 ± 0.00	565.08 ± 0.00
MAB_1839	AMK	2.91 ± 0.00	1.41 ± 0.22	0.61 ± 0.00	49.82 ± 59.10	3.59 ± 1.71	9.87 ± 4.05	3.19 ± 0.95	12.32 ± 6.63	1.48 ± 0.00
	CLA	0.70 ± 0.00	1.30 ± 0.00	0.24 ± 0.00	28.71 ± 39.15	1.99 ± 0.95	30.75 ± 15.17	3.12 ± 3.47	5.38 ± 5.94	23.59 ± 0.00
MAB_4036	AMK	0.28 ± 0.36	0.61 ± 0.22	0.22 ± 0.12	0.09 ± 0.01	0.00 ± 0.00	0.06 ± 0.02	0.04 ± 0.01	0.04 ± 0.01	1.53 ± 0.43
	CLA	4.90 ± 0.00	0.52 ± 0.00	0.67 ± 0.00	0.00 ± 0.00	0.01 ± 0.00	1.00 ± 0.00	0.12 ± 0.15	0.02 ± 0.01	0.62 ± 0.54
MAB_0734	AMK	17.07 ± 21.77	1.02 ± 0.00	0.50 ± 0.45	157.59 ± 26.79	27.17 ± 12.93	41.40 ± 16.98	72.91 ± 21.67	3.89 ± 3.37	0.42 ± 0.00
	CLA	19.76 ± 16.43	2.58 ± 0.00	2.91 ± 2.36	279.68 ± 193.33	33.19 ± 15.79	482.17 ± 237.95	47.47 ± 45.62	23.89 ± 23.00	370.44 ± 0.00
MAB_1171c	AMK	1.52 ± 0.56	1.36 ± 0.00	0.32 ± 0.11	4.50 ± 5.76	0.11 ± 0.05	0.23 ± 0.09	0.12 ± 0.04	1.19 ± 0.00	1.61 ± 1.35
	CLA	3.02 ± 0.00	1.14 ± 0.00	0.98 ± 0.42	1.00 ± 0.00	0.13 ± 0.06	0.31 ± 0.00	0.53 ± 0.72	0.45 ± 0.00	0.60 ± 0.00
MAB_1137c	AMK	13.65 ± 5.13	2.87 ± 2.40	2.86 ± 0.00	28.14 ± 10.27	3.00 ± 1.43	9.11 ± 3.74	7.58 ± 2.25	14.47 ± 13.00	0.57 ± 0.00
	CLA	6.54 ± 6.79	28.94 ± 36.87	0.05 ± 0.00	9.11 ± 7.59	0.77 ± 0.37	13.83 ± 6.83	2.15 ± 0.81	3.20 ± 2.60	33.55 ± 0.00

**MAB_2787c**	**AMK**	0.25 ± 0.16	3.27 ± 1.19	1.21 ± 0.00	0.25 ± 0.00	0.12 ± 0.06	1.52 ± 0.62	0.61 ± 0.18	0.06 ± 0.04	1.72 ± 1.36
	**CLA**	0.17 ± 0.00	1.46 ± 0.00	1.18 ± 0.00	0.22 ± 0.22	0.11 ± 0.05	0.24 ± 0.33	0.75 ± 0.88	0.35 ± 0.34	0.00 ± 0.00
**MAB_2421c**	**AMK**	0.40 ± 0.11	1.18 ± 0.25	0.45 ± 0.26	2.19 ± 1.94	0.21 ± 0.10	0.45 ± 0.18	1.54 ± 0.46	0.07 ± 0.05	0.22 ± 0.11
	**CLA**	0.39 ± 0.00	1.31 ± 0.15	0.65 ± 0.00	1.58 ± 1.67	0.12 ± 0.06	3.02 ± 1.49	0.87 ± 0.77	2.80 ± 2.86	1.11 ± 0.71
**MAB_3384c**	**AMK**	1.63 ± 0.00	0.69 ± 0.00	0.25 ± 0.16	2.56 ± 2.60	0.34 ± 0.16	0.64 ± 0.26	0.58 ± 0.17	0.27 ± 0.21	1.52 ± 0.83
	**CLA**	2.15 ± 0.00	0.21 ± 0.06	0.57 ± 0.00	2.01 ± 1.94	0.09 ± 0.04	2.80 ± 1.38	0.34 ± 0.31	0.20 ± 0.21	4.13 ± 5.42
**MAB_4117c**	**AMK**	0.20 ± 0.11	1.52 ± 0.09	1.09 ± 0.99	0.29 ± 0.34	0.08 ± 0.04	0.05 ± 0.02	0.09 ± 0.03	0.03 ± 0.00	1.24 ± 0.15
	**CLA**	6.29 ± 6.81	0.64 ± 0.01	2.26 ± 2.43	11.84 ± 4.09	2.58 ± 0.00	112.72 ± 92.50	2.50 ± 3.50	5.89 ± 0.00	3.16 ± 2.55
**MAB_3465**	**AMK**	14.57 ± 3.69	0.66 ± 0.15	0.31 ± 0.18	16.23 ± 12.00	2.26 ± 1.07	6.76 ± 2.77	5.85 ± 1.74	0.63 ± 0.36	0.92 ± 0.43
	**CLA**	22.78 ± 25.20	0.60 ± 0.40	0.63 ± 0.01	18.93 ± 13.00	1.26 ± 0.60	21.66 ± 10.69	6.08 ± 6.87	20.93 ± 20.50	38.37 ± 28.22
**MAB_4915c**	**AMK**	1.20 ± 0.41	0.76 ± 0.21	0.46 ± 0.40	0.28 ± 0.16	0.30 ± 0.14	0.28 ± 0.11	0.65 ± 0.19	0.30 ± 0.35	1.59 ± 0.62
	**CLA**	1.11 ± 0.65	1.52 ± 0.00	1.66 ± 0.02	1.07 ± 1.16	0.14 ± 0.07	6.15 ± 3.03	0.44 ± 0.43	0.09 ± 0.04	2.61 ± 2.56
